# Identification of WT1 as determinant of heptatocellular carcinoma and its inhibition by Chinese herbal medicine *Salvia chinensis* Benth and its active ingredient protocatechualdehyde

**DOI:** 10.18632/oncotarget.22406

**Published:** 2017-11-11

**Authors:** Ning Wang, Hor-Yue Tan, Yau-Tuen Chan, Wei Guo, Sha Li, Yibin Feng

**Affiliations:** ^1^ School of Chinese Medicine, The University of Hong Kong, Pokfulam, Hong Kong S.A.R., China

**Keywords:** hepatocellular carcinoma, WT1, Wnt/β-catenin pathway, Chinese herbal medicine, molecular docking-guided bioactive ingredient identification

## Abstract

Candidates from Chinese herbal Medicine might be preferable in drug discovery as the abundant experiences of traditional use usually hint the clinical efficacy. In this study, we screened the anti-tumour effect of several commonly used Chinese herbal Medicines on human hepatocellular carcinoma cells (HCC). We identified that Salvia chinensia Benth. (Shijianchuan in Chinese, SJC) exhibited prominent *in vitro* inhibition of HCC cells and suppressed the orthotopic growth of HCC in the liver of mice and repressed the lung metastasis of tumour cells. Using a pathway-specific PCR array and Gene Ontology analysis, we identified that Wnt/β-catenin pathway was associated with the suppressive effect of SJC on HCC cell proliferation and cell cycle progression. SJC repressed transcription activity of Wnt/β-catenin pathway and reduced expression of β-catenin in GSK-3β-independent but promoter-specific transcription inhibition mechanism. The suppressive effect of SJC on β-catenin expression and its transcription activity was associated with Wilms’ tumor 1 (WT1) protein. WT1 was overexpressed in HCC tissues, and was negatively correlated to the overall survival of HCC patients. WT1 promoted proliferation and invasion of HCC cells, as well as β-catenin-dependent transcription activation of Wnt products, while knockdown of WT1 had the opposite effect. Docking experiment revealed that protocatechualdehyde (PCA) might be the active component of the herb. PCA suppressed transcription activity of Wnt/β-catenin pathway in WT1-dependent manner. Our study sheds light on the potential of PCA from commonly used anti-cancer Chinese herbal Medicine SJC as a lead compound targeting WT1 in the discovery of anti-HCC drugs.

## INTRODUCTION

Hepatocellular carcinoma is one of the most malignant human cancers that results in increasing annual cancer death, ranking the second highest mortality rate all over the world [1]. Although a few of HCC patients may be suitable for surgical resection and liver transplantation which gain satisfactory therapeutic outcome [2], a large number of patients suffering unresectable HCC can only be given treatments such as transcatheter arterial chemoembolization (TACE) and sorafenib, whose outcome may be less satisfactory [3]. A recent study analysing multiple levels of comprehensive genomic characterization of HCC suggested Wnt signalling is one of the potential targets of next generation treatments [4]. Activation of Wnt signalling is involved in various processes of HCC development, and β-catenin, which is encoded by human CTNNB1 gene, plays a central role in mediating the hyper-activation of Wnt signalling during hepatic carcinogenesis and cancer progression [5]. Overexpression of β-catenin was found in HCC cases [6], and it is believed that ablation of epigenetic and genetic control of this gene is the leading cause of overexpression [7]. Approved target-specific therapies on Wnt/β-catenin signalling are yet not available, however, some studies have shown that inhibition of Wnt/β-catenin can improve the sensitivity of HCC in response to sorafenib [8, 9], as Wnt/β-catenin signalling was found to mediate the mechanism of resistance of HCC cells towards sorafenib treatment [10, 11].

Some of the Chinese herbal Medicines have been widely reported for their inhibitory effect on HCC at pre-clinical and clinical levels [12, 13]. It has been summarized by other's and our labs that a list of Chinese herbal Medicines is commonly used in the clinical treatment of HCC by physicians, including some of which were rarely studied in scientific extent [13–16]. Although clinical experience on the use of these Chinese herbal Medicines might hint its therapeutic potential, scientific evidences underlying its anti-tumour activity was scanty. On the other hand, identification of novel anti-tumour lead compounds from herbal Medicine, as successful as the cases of paclitaxel and camptothecin [17], requires both understandings on the disease targets and chemical compositions of the complicated herbal extracts. The integration of experimental validation with computational data mining is a helpful approach to overcome the technology barriers in identifying target-specific and active molecules from Chinese herbal Medicine. Recently, a study has shown the powerfulness of combining molecular docking with experimental verification in fishing the active ingredients of Chinese Herbal Remedy Free and Easy Wanderer in treating mental disorder [18]. The successful application of this study pattern suggested its potential application in efficient drug discovery from Chinese herbal Medicine [19].

In this study, we first of all screened several rarely studied Chinese herbal Medicines that have been extensively used in clinical treatment of HCC by Chinese Medicine practitioners. We identified that Salvia chinensia Benth. (Shijianchuan in Chinese, **SJC**) was the most prominent suppressor of HCC growth. We systemically evaluated the *in vitro* and *in vivo* anti-HCC activity of SJC, and identified by disease-specific array that WT1-associated Wnt/β-catenin was involved in the pharmacological action of SJC. As a novel oncogene in HCC, the clinical significance and biological function of WT1 was determined in this study. In addition, prediction and validation of biologically active ingredient of SJC was performed using computational molecular docking combined with bioassay validation.

## RESULTS

### Aqueous extract of Salvia chinensia Benth. exhibited potent inhibition on *in vitro* proliferation and viability of HCC cells

**Figure 1:**
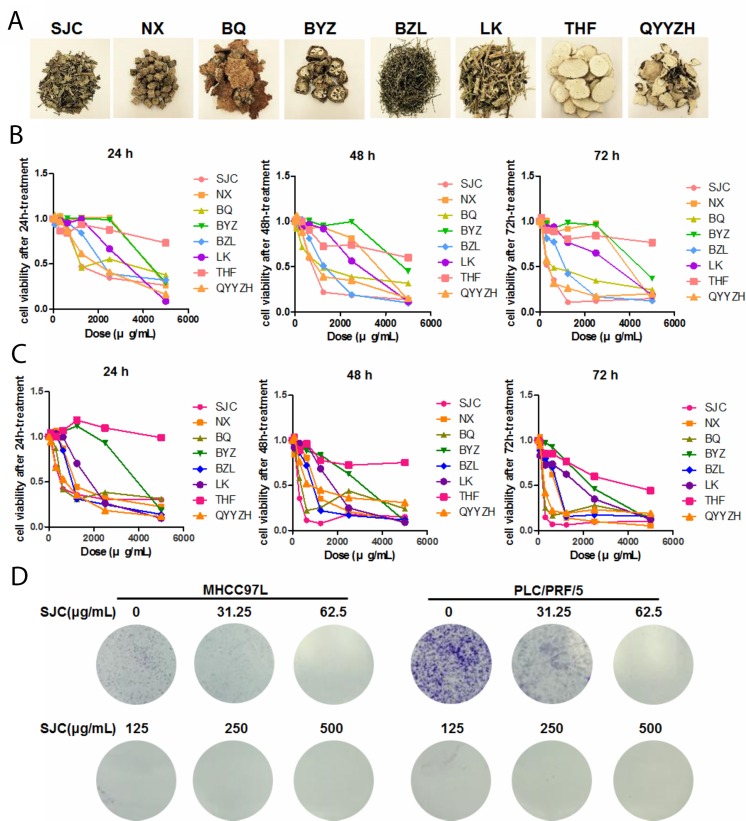
SJC suppressed hepatocellular carcinoma cells *in vitro* **(A)** representative images of eight commonly used Chinese herbal Medicines in the clinical treatment of HCC; **(B)** cytotoxicity of aqueous extract of Chinese herbal Medicines on human HCC cell line MHCC97L; **(C)** cytotoxicity of aqueous extract of aforementioned Chinese herbal Medicine on human HCC cell line PLC/PRF/5; **(D)** SJC suppressed growth of MHCC97L and PLC/PRF/5 cells, as indicated by colony formation assay.

A plenty of Chinese herbal Medicines are commonly prescribed to patients with HCC in the clinical practice of Chinese Medicine practitioners [13]. Some were extensively studied in the past decades, such as Coptis chinensis Franch. (Huanglian in Chinese), Oldenlandia diffusa (Willd.) Roxb (Baihua Sheshe Cao in Chinese), Sophora flavescens Ait. (Kushen in Chinese) and Radix ginseng (Renshen in Chinese). Here we selected eight less studied anti-HCC Chinese herbal Medicines to screen the *in vitro* cytotoxicity (Figure 1A). Aqueous extract of each individual herb was made and the *in vitro* cytotoxicity towards human HCC cells PLC/PRF/5 and MHCC97L was measured by MTT assay. It was shown that among the eight herbs, Salvia chinensia Benth. (Shijianchuan in Chinese, SJC, chromatographic fingerprint at Supplementary Figure 1) exhibited the most potent inhibition on the viability of HCC cells (IC50 equal to 500, 250 and 200μg/mL at 24, 48 and 72 h-treatment in PLC/PRF/5 cells, and equal to 1000, 600 and 300μg/mL at 24, 48 and 72 h-treatment in MHCC97L cells, Figure 1B&1C). Paris polyphylla (Qiye Yizhi Hua in Chinese, QYYZZ) and Smilax china L. (Baiqia in Chinese, BQ) possessed similar cytotoxicity with SJC at 24 to 48 h, while HCC cells quickly acquired adaptation which rendered resistance to longer treatment of QYYZZ and BQ. Inhibition of clony formation of both HCC cells by a 12-day constitutive treatment with SJC further proved the long-term inhibitory effect of SJC extract on HCC cells without obvious resistance observed.

### Oral administration of SJC extract suppressed *in vivo* proliferation and metastasis of HCC

**Figure 2:**
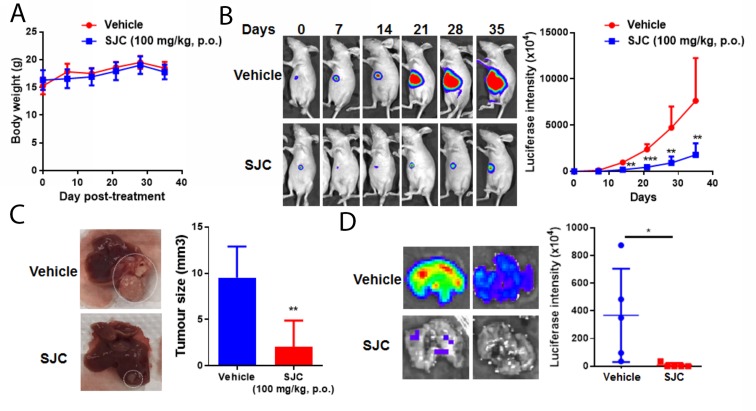
SJC exhibited anti-tumour effect in orthotopic HCC implantation model **(A)** shows oral administration of SJC had minimal effect on the body weight of athymic nude mice; **(B)** shows SJC can regress orthotopic growth of implanted HCC, as indicated by luciferase reporter-dependent live animal imaging; reduced signal intensity in SJC-treated mice reveals smaller tumour size; **(C)** shows representative images of HCC in the liver of mice; **(D)** shows that SJC suppressed distant metastasis of HCC towards lung tissue, as indicated by bare-to-none luciferase signal in the lungs of SJC-treated mice.

To further examine the clinically relevant anti-HCC effect of SJC, we established the orthotopic HCC implanted model in nude mice. Luciferase reporter was transfected into MHCC97L, the cell line that was then used to generate a small cube of solid tumour being implanted to the left lobe of receiving mice. Body weight measurement revealed that oral treatment of SJC extract (100 mg/kg/day) had minimal effect on body weight, indicating no observational toxicity of the herb (Figure 2A). *in vivo* imaging showed that SJC treatment repressed the orthotopic tumour growth of HCC. While mice receiving vehicle treatment gained enlarging hepatic tumour, the increase in tumour size and reporter signal intensity in SJC-treated mice was not potent (Figure 2B). This was complying with the end-point observation, which showed that mice with vehicle treatment had a larger liver tumour than mice treated with SJC (Figure 2C). This proved the *in vivo* inhibitory effect of SJC on HCC proliferation and growth. Furthermore, as lung metastasis was one of the indicators of HCC aggressiveness [20], we measured if SJC treatment could block the extrahepatic spread of HCC cells in mice. Dissected lung from mice with vehicle treatment exhibited strong luciferase signal, indicating a severe lung metastasis of HCC cells; weak to none signal could be detected in lung from mice with SJC treatment, suggesting that the lung metastasis was completely blocked. Our findings suggested that SJC suppressed *in vivo* proliferation and distant metastasis of HCC without exhibiting obvious toxicity.

### SJC intervention might impede cell proliferation and cell cycle progression and block canonical Wnt pathway

**Figure 3:**
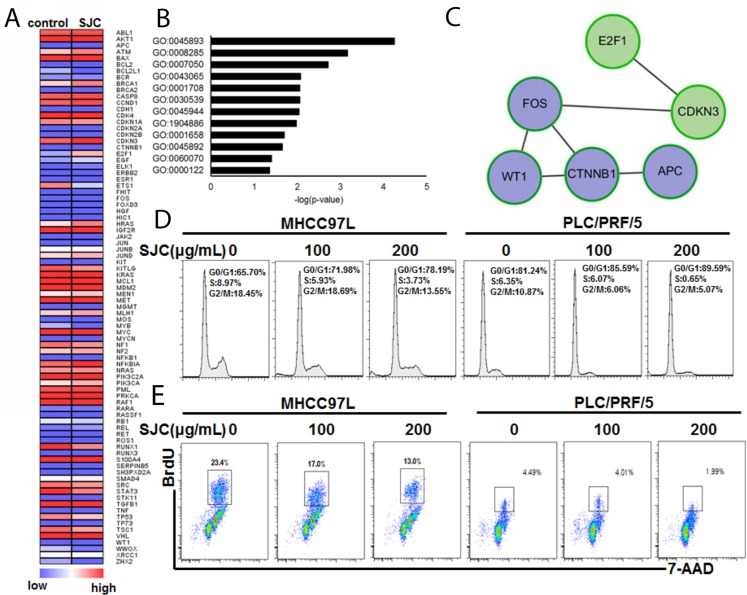
SJC caused multiple changes of gene expression that are involved in cell cycle and proliferation regulation **(A)** heatmap of analysis on the PCR pathway array. SJC treatments induced expression change of a series of genes in MHCC97L; **(B)** GO analaysis on the genes with differential expression. It was found that Wnt/β-catenin pathway was enriched with most significant p-value; **(C)** genes in Wnt/β-catenin pathway that was regulated by SJC treatment; **(D)** SJC led to G0/G1 cell cycle arrest in HCC cells; **(E)** SJC treatment inhibited population of HCC cells with rapid proliferation (S phase).

To make a global understanding on the pharmacological effect of SJC, we introduced HCC-specific pathway PCR array. A series of genes were regulated by SJC in HCC cells, nine of which were down-regulated while six were induced (Figure 3A, Supplementary Table 2). Enrichment on the biological process that the regulated genes were involved in showed that SJC could interfere cellular process related to DNA transcription (GO:0045893), cell proliferation (GO:0008285) and cell cycle progression (GO:0007050), all of which might contribute to the rapid growth of liver tumour (Figure 3B, Supplementary Table 3). Interestingly, it was noticed that the action of SJC might also involve regulation on the canonical Wnt pathway (GO:0060070). We further analysed the involving genes into String database to figure out the potential interactive network underlying SJC's pharmacological action. The protein network constructed by Cytoscape further confirmed that a series of members of Wnt/β-catenin pathway were involved (Figure 3C), which covered from the upstream co-regulator APC, executor β-catenin and effector c-fos. In addition, genes in the regulation of cell cycle might be also involved. To validate the prediction biological process, we analysed the cell cycle progression and proliferation of HCC cells upon SJC treatment. PI staining of cellular DNA revealed that SJC could halt the cell cycle at G0/G1 stage (Figure 3D). BrdU incorporation assay showed that the proliferation rate of HCC cells was slowed down by SJC treatment (Figure 3E). These findings suggested that the pharmacological action of SJC was associated with the regulation of cell proliferation and cell cycle progression via Wnt/β-catenin pathway.

### SJC suppressed Wnt/β-catenin pathway in WT1-dependent and GSK3β-independent manner

**Figure 4:**
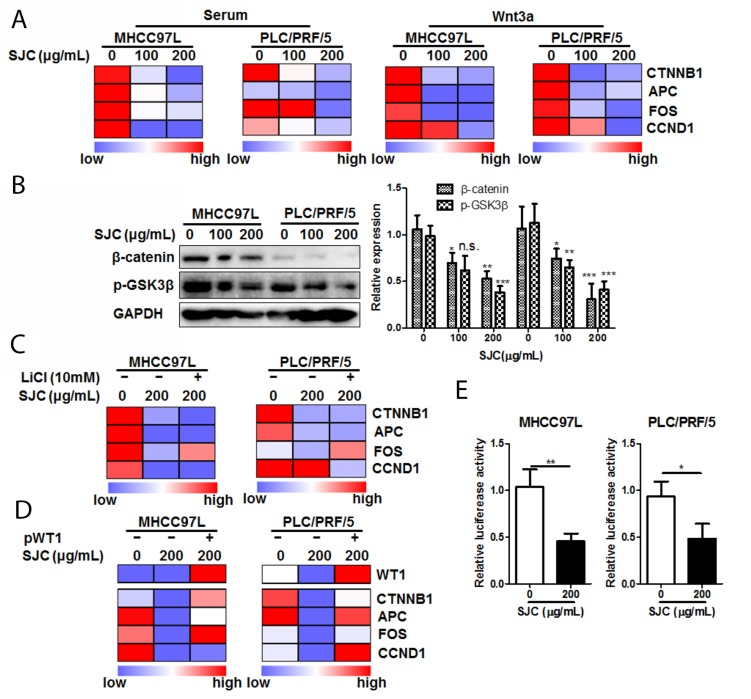
SJC suppressed Wnt/β-catenin pathway activity via regulating WT1-associated β-catenin transcription **(A)** SJC suppressed transcription of Wnt/β-catenin products in HCC cells, indicating repression of Wnt/β-catenin pathway activity by SJC; **(B)** SJC suppressed expression of β-catenin without altering activity of GSK3β; **(C)** inhibition of GSK3β by LiCl cannot attenuate inhibition of Wnt/β-catenin pathway activity by SJC; **(D)** overexpression of WT1 restored expression of β-catenin as well as the transcription activity of Wnt pathway; **(E)** SJC possessed promoter-specific transcription inhibition mechanism on β-catenin expression.

To further understand the action and action of mechanism underlying regulation of Wnt/β-catenin pathways by SJC, we exposed HCC cells with SJC in the presence or absence of Wnt3a, a stimulator of canonical Wnt pathway. Treatment of SJC could significantly suppress the expression of Wnt pathway product genes (Figure 4A). The inhibitory effect of SJC on transcription of Wnt pathway products could sustain during the treatment, and re-exposure of Wnt3a for 6 h after SJC treatment cannot recover the transcription activity of Wnt signalling. Immnoblotting analysis showed that SJC treatment substantially suppressed expression of β-catenin, whose expression and protein stabilization are required for the Wnt activation and subsequent gene transcription [21]. SJC had minimal effect on the phosphorylation of GSK3β, the internal inhibitor of β-catenin by mediating its phosphorylated degradation [22] (Figure 4B). This observation together with the finding that the mRNA transcripts of β-catenin were reduced in SJC-treated HCC cells (Figure 4A) indicated a posttranslational regulation-independent mechanism may be involved. Suppression of β-catenin expression by SJC cannot be attenuated by GSK3β inhibitor LiCl, further proving that the inhibitory effect of SJC on β-catenin expression was independent to GSK3β (Figure 4C). Notably, the constructed protein network underlying pharmacological action of SJC and Immunoblotting analysis revealed the involvement of Wilms’ tumor protein (WT1) in the regulatory network of Wnt/β-catenin pathway. To examine if WT1 was involved in the regulation of β-catenin by SJC, we re-expressed WT1 in SJC-treated HCC cells. Recovery of WT1 expression was confirmed by qPCR, and WT1 re-expression could restore the mRNA transcription of Wnt pathway products as well as β-catenin expression (Figure 4D). Luciferase reporter assay also revealed that suppression of β-catenin transcription by SJC was partially recovered by re-expression of WT1 in HCC cells (Figure 4E). Our findings suggested that SJC inhibited Wnt/β-catenin pathway in WT1-dependent manner.

### WT1 was overexpressed in HCC and facilitated proliferation and invasion of HCC cells

**Figure 5:**
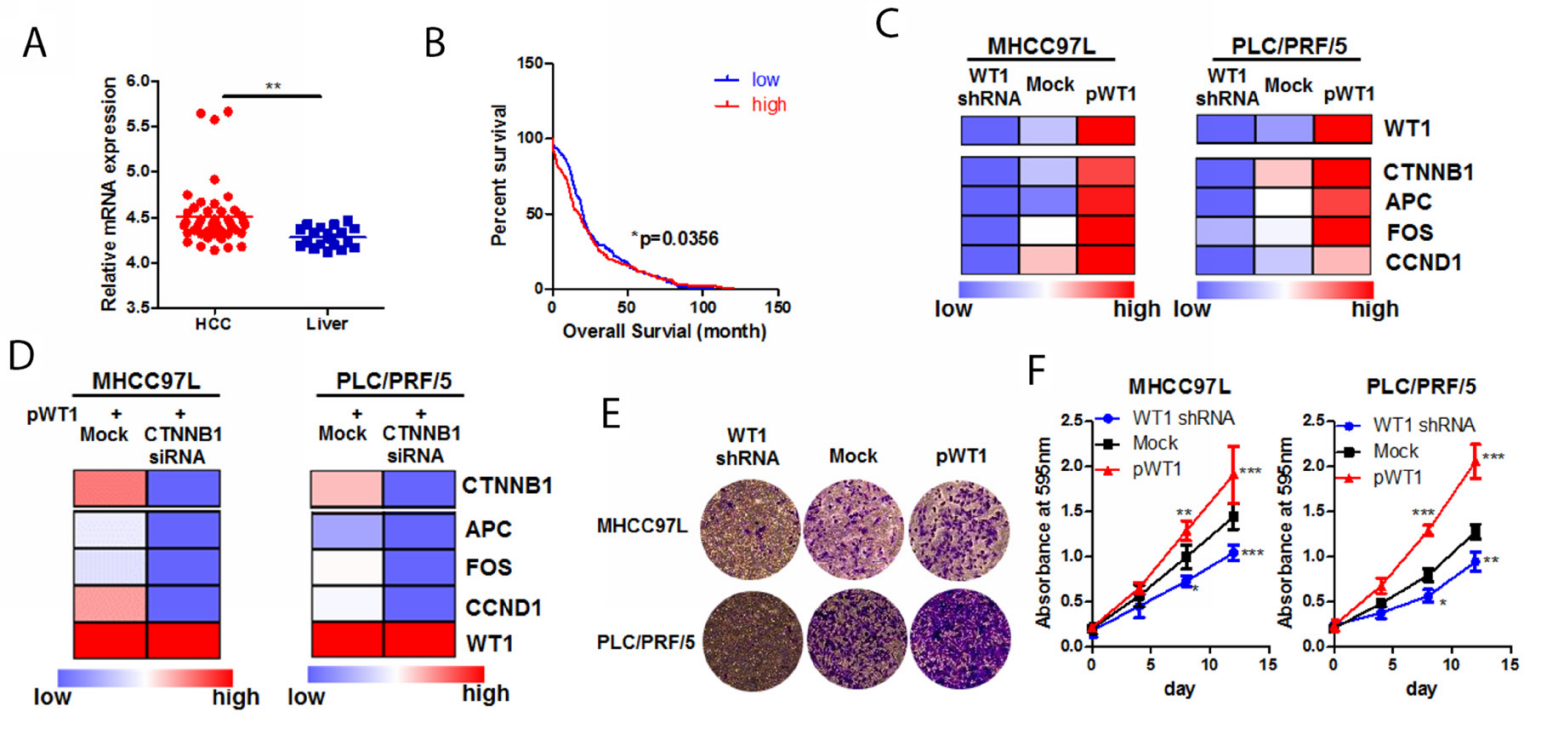
WT1-promoted HCC progression was associated with Wnt/β-catenin pathway **(A)** mining of GSE14323 dataset revealed that WT1 was overexpressed in HCC tissue compared with non-tumour liver; **(B)** high expression of WT1 was associated with poor survival in HCC patients. Data was mined from TGCA database and KM plot was created by grouping survival data of patients with WT1 mRNA expression higher than 1.3 from those lower than 1.3; **(C)** WT1 regulated activity of Wnt/β-catenin pathway; HCC cells overexpressing WT1 exhibited increased production of Wnt/β-catenin targets, while knockdown of WT1 expression repressed transcription of Wnt/β-catenin products; **(D)** knockdown of β-catenin abrogated WT1-induced Wnt/β-catenin pathway; **(E)** overexpression of WT1 promoted HCC cell invasion while WT1 knockdown blocked invasion of HCC cells. Expression of WT1 was shown in **c**; **(F)** overexpression promotes HCC cell proliferation while WT1 knockdown retarded its growth. Expression of WT1 was shown in **c**.

Although some previous studies have shown that WT1 expression in HCC was associated with its poor prognosis [23], the clinical-relevant biological function of WT1 on HCC has not been fully unveiled. To further evaluate the potential of WT1 as a possible drug target in HCC treatment, we analysed the clinical significance of WT1 expression in HCC by extracting GEO database GSE14323 (https://www.ncbi.nlm.nih.gov/geo/query/acc.cgi?acc=GSE14323). WT1 was significantly overexpressed in HCC tissue in compared with non-tumour liver (Figure 5A). KM plot analysis on survival data of HCC patients showed that patients with higher expression of WT1 in tumour (greater than 1.3 FPKM, data from transcriptomic sequencing of TGCA database) exhibited lower median survival than those with lower expression of WT1 (less than 1.3 FPKM; Figure 5B, p=0.0356). These may suggest high expression of WT1 in HCC would link to poor survival outcome. To further understand the biological function of WT1, we overexpressed and knock-downed WT1 in HCC cells. Expression of Wnt pathway products was regulated upon the genetic modification of WT1 in HCC cells. Overexpression of WT1 could increase transcription of Wnt pathway products while silence of WT1 suppressed transcription activation upon Wnt3a stimulation (Figure 5C). To examine if the activation of Wnt pathway by WT1 was dependent on β-catenin, we interfered β-catenin in WT1-overexpressing HCC cells. Knockdown of β-catenin in WT1-overexpressing HCC cells repressed expression of Wnt pathway products, which suggested the necessary role of β-catenin in mediating WT1-induced Wnt activation (Figure 5D). Functional assays revealed that overexpression of WT1 could promote the proliferation and invasion of HCC cells, while knockdown of WT1 impeded HCC cells (Figure 5E&5F). These findings suggested high expression of WT1 promoted HCC proliferation and invasion with involvement of Wnt activation.

### Protocatechualdehyde (PCA) from SJC was responsible for the inhibition of WT1-associated Wnt activation

**Figure 6:**
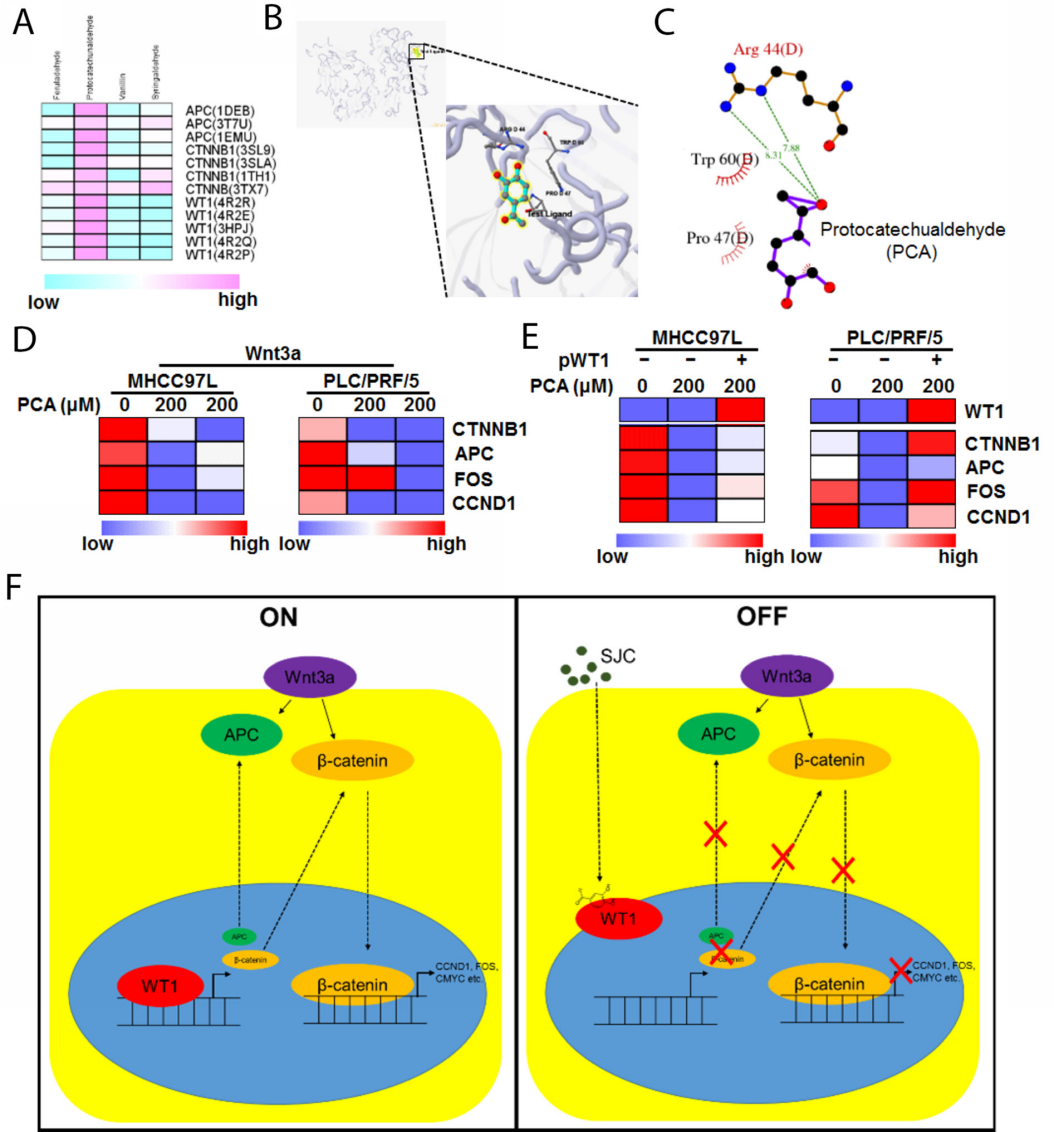
PCA may be the active ingredient of SJC in suppressing HCC **(A)** shows the binding of potential of compounds in SJC with possible molecular targets. Only PCA showed active binding to the proteins in which WT1 exhibited higher binding score with PCA; **(B)** shows the 3D image of binding profile between WT1 protein and PCA; **(C)** shows residues of WT1 protein that can interact with PCA; **(D)** PCA suppressed transcription of Wnt/β-catenin products in HCC cells; **(E)** overexpression of WT1 restored the transcription activity of Wnt/β-catenin pathway in PCA-treated cells; **(F)** the schematic representative of regulatory mechanism underlying WT1-mediated inhibition of HCC by SJC.

To further understand the molecular basis of the anti-HCC action, we tried to explore the possible active components of SJC with combination of computational and experimental approaches. First, we searched for chemical ingredients SJC on Traditional Chinese Medicine Systems Pharmacology Database (TCMSP, http://lsp.nwsuaf.edu.cn/). Compounds with favourable pharmacokinetic properties (OB ≥ 33% and Caco2 ≥ 0.4 cm/s) were selected. Five compounds (PCA, vanillin, syringaldehyde, ferulaldehyde and β-sitosterol) were shortlisted. As β-sitosterol has wide distribution in a lot of natural plants and may not be a specific component of SJC, only four phenolic compounds were performed molecular docking analysis with particular structure of several proteins identified in the regulatory network of Figure 3B. Among the Wnt proteins, c-fos exhibited no interaction with the input compounds, while APC, β-catenin and WT1 had less binding capacity with anillin, syringaldehyde and ferulaldehyde (Figure 6A). Using 5.22 as a threshold of active binding score, only PCA was qualified to actively interact with the selected proteins, among which WT1 exhibited the highest binding potency with PCA (Figure 6B), and three residues of WT1, Arg44, Trp60 and Pro47 formed a pocket for the binding of PCA (Figure 6C). Reduced expression of Wnt pathway products upon PCA treatment was experimentally observed (Figure 6D), and overexpression of WT1 attenuated this inhibitory effect (Figure 6E), confirming that the inhibitory effect of PCA on Wnt activation was associated with WT1 expression in HCC cells.

## DISCUSSION

The inhibitory effect of SJC has ever reported by a previous study with some individual signalling pathways were examined [24]. A global screening may be necessary for identifying the true target of SJC in treating HCC. By applying PCR pathway array, we tried to elaborate the pharmacological action of Chinese herbal Medicine in the throughout approach. Although SJC extract may contain a series of compounds in different structure and bioactivity, the overall outcome of regulation on cellular signalling by such a complicated mixture shall be identifiable by means of detection of changes on global gene profile [25]. Assays may differ in terms of throughput level, but outcome of analyses shall be identical. In our study, by visiting the differential gene expression, we identified that Wnt pathway was majorly involved in the action of SJC on HCC cells.

The inhibitory effect of PCA on human tumour cells was reported in a few previous studies. It was particularly noticed by Jeong and colleague that PCA can potently inhibit growth of colorectal cancer cells with suppression of HDAC2 and Cyclin D1 expression. To be sepcific, PCA suppressed Cyclin D1 at transcriptional level, as PCA reduced both Cyclin D1 promoter activity and mRNA expression in colorectal cancer cells [26]. Further studies supported the notion that PCA-induced Cyclin D1 inhibition may subsequently cause cell cycle arrest and apoptosis of various human cancer cells [27, 28]. Noticeably, a recent study revealed that PCA-suppressed Cyclin D1 can be dependent on Wnt/β-catenin pathway. The effect of PCA on the pathway can be partially dependent to the protein stability of β-catenin, but more importantly, to nuclear translocation of NF-κB, suggesting a NF-κB-associated transcriptional inhibition mechanism of β-catenin might be involved and NF-κB may play as a transcription repressor of β-catenin [29]. In our study we found that WT1 can positively regulate Wnt pathway activity through promoting β-catenin transcription. While previous study has demonstrated that NF-κB did not regulate WT1 expression [30], it may be postulated that WT1 and NF-κB may have interaction with β-catenin promoter. As WT1 and NF-κB was found to physically interact with each other in mammalian cells [31], the WT1-mediated β-catenin may be probably through recruiting NF-κB to form protein complex and therefore releasing β-catenin promoter from NF-κB-mediated suppression on transcription initiation.

We observed that the effect of SJC on Wnt/β-catenin signalling was associated with WT1 in HCC cells. An integrated genome-wide screening have identified that the activity of Wnt/β-catenin signalling is associated with WT1 expression [32]. An earlier study has mapped the promoter region of Wnt-4, ligand of Wnt pathway. It was found that though Wnt-4 promoter presents several binding site of WT1, the regulation of WT1 on Wnt-4 expression may be indirect [33]. However, Schittenhelm and colleague reported that there was no evidence to show that WT1 was involved in the regulation of non-canonical Wnt pathway which was mediated by Wnt-4/β-catenin [34]. Knockout of WT1 reduced expression of canonical Wnt pathway elements LEF1 and CTNNB1, suggestive of diminished canonical Wnt signalling [35]. It was also found that in testis cells, knockdown of WT1 can reduce expression of Lef1 and Ptch1, indicating suppression of Wnt signalling [36]. These observations in accordance with our findings may reveal that WT1 can regulate the canonical instead of non-canonical pathway of Wnt signalling. Furthermore, in our study, with luciferase reporter assay we found the transcriptional regulation of SJC onβ-catenin expression, however, we cannot directly conclude that WT1 serves as transcription factor that binds to CTNNB1 promoter region. Indeed, a recent study revealed that WT1 can repress EZH2-mediated epigenetic silencing of CTNNB1 gene, and therefore promote β-catenin expression [37]. The transcriptional regulation of WT1 on β-catenin may thus be indirect and is dependent to the presence of other determinant factors. This may also explain the observations that in some circumstances, WT1 may serve as a repressor of β-catenin [38–41]. The exact mechanism of WT1-regulated Wnt/β-catenin activity needs further investigation.

In this study, we identified the anti-tumour activity of a clinically used Chinese herbal Medicine Salvia chinensia Benth. (Shijianchuan in Chinese) in HCC. We screened and identified that SJC was one of most prominent herbs that possessed inhibition on HCC. SJC exhibited cytotoxicity in HCC cells, and suppressed the *in vitro* proliferation and cell cycle progression. In mice with orthotopic implanted HCC, oral treatment of SJC reduced tumour growth and lung metastasis. Disease-specific PCR array and GO analysis showed that Wnt/β-catenin was associated with the inhibitory effect of SJC, and WT1 might be the up-stream molecule that regulated Wnt/β-catenin activity. Treatment of SJC suppressed Wnt/β-catenin activity via repressing β-catenin transcription in HCC cells, which was independent to GSK-3β pathway but could be attenuated by WT1 overexpression. As a novel identified oncogene, WT1 was found overexpressed in human HCC compared with non-tumour liver, and its high expression correlates with low overall survival. Overexpression of WT1 promoted proliferation and invasion of HCC cells, and evoked Wnt/β-catenin signalling in β-catenin-dependent manner. Protocatechualdehyde was predicted to be the possible active ingredient in SJC by computational annotation and molecular docking. Suppression of Wnt/β-catenin signalling by protocatechualdehyde was attenuated by WT1 overexpression. Our study suggested that WT1-regualted Wnt/β-catenin signalling may be a potential target of HCC treatment, and SJC and its active ingredient protocatechualdehyde may be possible for development of new treatment targeting WT1.

## MATERIALS AND METHODS

### Herbal extract preparation

Dried herbs of eight Chinese herbal Medicines, including Achyranthes bidentata Bl. (Niuxi in Chinese, **NX**), Salvia chinensia Benth. (Shijianchuan in Chinese, **SJC**), Fruit of Fiverleaf Akebia (Bayuezha in Chinese, **BYZ**), Portulaca Grandiflora (Banzhilian in Chinese, **BZL**), Solanum nigrum (Longkui in Chinese, **LK**), Radix Trichosanthis (Tianhuafen in Chinese, **THF**), Paris polyphylla (Qiye Yizhi Hua in Chinese, **QYYZH**) and Smilax china L. (Baqia in Chinese, **BQ**), were collected from the clinical centre of School of Chinese Medicine, The University of Hong Kong. All the herbs were authenticated by Dr. Feng Yibin. Species of individual herb were kept and voucher numbers were given. To obtain the aqueous extracts of Chinese herbal Medicines, minced herb was boiled twice with 10 times of water for 2 h, follow by freeze-dry of the extracts. The dry powder was kept in −20°C and reconstituted in appropriate vehicle before use. Doses used in this study were presented in equal weight of raw herb per millilitre of vehicle.

### Cell lines and cell culture

Human hepatocellular carcinoma cell line PLC/PRF/5 was obtained from ATCC (USA). MHCC97L cells expressing luciferase reporter gene were kindly gifted by Prof. Man Kwan in Department of Surgery, The University of Hong Kong. All cells were maintained by Dulbecco's Modified Eagle's Medium (DMEM, high glucose) supplemented with 10% Fetal Bovine Serum (FBS) and 1% Penicillin/Streptomycin in humidified condition with 5% CO_2_ at 37°C.

### siRNA, plasmid and transfection

siRNA against human β-catenin was purchased from Santa Cruz. pAd/WT1-IRES-nAmCyan was a gift from Edward McCabe (Addgene plasmid # 29756). shRNA against WT1 and β-catenin promoter reporter were from Genecopoeia (USA). Transfection was conducted with FuGene Transfection reagent (Promega, USA). In brief, corresponding nucleotide was mixed with FuGene reagent in serum free medium (1:2, w/v) followed by 15 min incubation at room temperature. The mixture was then added to the cells and incubated for 24 to 72 h. Treatment was applied during the incubation if necessary.

### Orthotopic HCC implantation in athymic nude mice

Protocol of animal experiment has been approved by the Committee on the Use of Live Animals in Teaching and Research (CULATR) of the University of Hong Kong (Ref: 3776-15). Orthotopic HCC implantation was performed on 6-week nude mice with surgical procedure reported by our previous study [42]. In brief, solid tumour was formed by subcutaneously injecting luciferase-tagged MHCC97L cells into the right flank of athymic nude mice. Small cube of tumour was then cut and implanted into the left lobe of liver of receiving mice. 7 days after implantation, mice were given SJC treatment (100 mg/kg/day) via oral gavage for 5 weeks. Growth of hepatic tumour was monitored weekly under IVIS live animal imager. At the end of study, mice were sacrificed by overdose pentobarbital (200 mg/kg) and liver and lung were dissected out for imaging.

### RNA extract, PCR array and real-time PCR

Total RNA was extracted using RNeasy mini kit (Qiagen, Germany) under manufacturer's instruction. First strand cDNA was prepared with RT^2^ First Strand Kit (Qiagen, Germany). For PCR array, the pre-coated Liver Cancer RT2 Profiler kit (PAHS-133Z, Qiagen, Germany) was used; for real-time PCR, cDNA was amplified in SYBR Green I reagent (Takara, Japan) with specific primer sets (Supplementary Table 1) for particular genes. All the assays were performed on LC480 platform (Roche, USA).

### Flow cytometry

For analysis of cell cycle, synchronized cells were treated with SJC and fixed in 70% Ethanol, followed by propidium iodide (PI, 50 μg/mL, Sigma-aldrich, USA) staining in dark for 40 min. For analysis of cell proliferation, SJC-treated cells were incubated with BrdU reagents for 1 hr. Staining was performed with FITC BrdU Flow Kit (BD Bioscience, USA) according to manufacturer's instruction. Cell cycle and proliferation were then analysed on Canto II flow cytometer (BD Bioscience, USA).

### Luciferase reporter assay

Cells transfected with GLuc-ON containing promoter region of human β-catenin (Genecopeia, USA) were treated with SJC for 48 h. Cells were then lysed and the firefly luciferase activity was detected. The lysate was detected by adding 150 μg/mL luciferin (Promega, USA) as substrate and measuring the signal intensity on IVIS imager.

### Computational modelling

List of ingredients in SJC was extracted from on Traditional Chinese Medicine Systems Pharmacology Database (TCMSP, http://lsp.nwsuaf.edu.cn/). Only compounds which meet the criteria of oral availability (OB) ≥ 33% and predicted permeability on Caco2 cells (Caco2) ≥ 0.4 cm/s were shortlisted. Structure of the shortlisted compounds as well as protein PDB IDs were input into the on-line docking software systemDocks (http://systemsdock.unit.oist.jp/iddp/home/index/). Binding portfolio of compound-protein pairs was shown when the predicted active binding score was larger than default threshold (5.22 in the case).

### Statistical analysis

Results were analysed using One-way ANOVA and expressed as mean ± SD.

## SUPPLEMENTARY MATERIALS FIGURE AND TABLES


