# Supplementary material for: Identification of WT1 as determinant of heptatocellular carcinoma and its inhibition by Chinese herbal medicine Salvia chinensis Benth and its active ingredient protocatechualdehyde
Source: Oncotarget. 2017 Nov 11;8(62):105848–59. doi: 10.18632/oncotarget.22406 (PMC5739684; doi:10.18632/oncotarget.22406)
Supplement: Supplementary file 1 [file oncotarget-08-105848-s001.pdf]

Identification of WT1 as determinant of heptatocellular carcinoma and its inhibition by Chinese herbal medicine *Salvia chinensis* Benth and its active ingredient protocatechualdehyde

SUPPLEMENTARY MATERIALS

Supplementary Table 1: Primer sequence for qPCR

Gene	Sequence
wt1	*5'-GATAACCACACAACGCCCATC-3'*
	5'-CACACGTCGCACATCCTGAAT-3'
Apc	*5'-CCTCATCCAGCTTTTACATGGC-3'*
	5'-CGCCTGCCTCTCTTGTCAT-3'
ctnnb1	*5'-AAAATGGCAGTGCGTTTAG-3'*
	5'-TTTGAAGGCAGTCTGTCGTA-3'
fos	*5′-CTGGCGTTGTGAAGACCAT-3′*
	5′-TCCCTTCGGATTCTCCTTTT-3′
ccnd1	*5'-AAGGCGGAGGAGACCTGCGCG-3'*
	5'-ATCGTGCGGCATTGCGGC-3'
β-actin	*5'-GCTTCTCCTTAATGTCACGC-3'*
	5'-CCCACACTGTGCCCATCTAC-3'

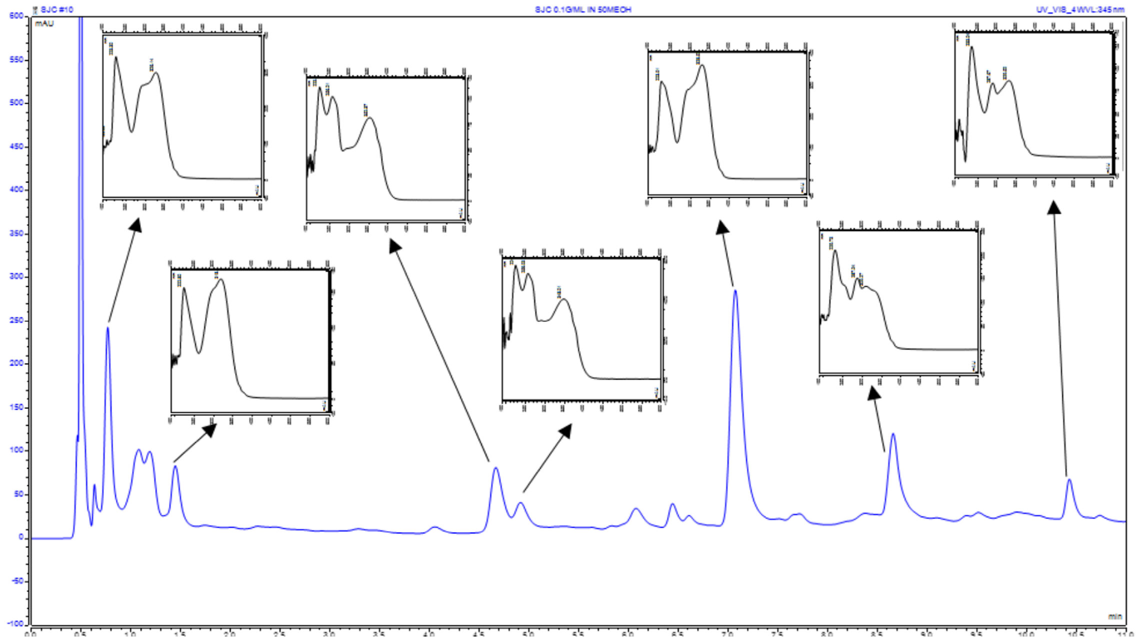

Supplementary Figure 1: Chromatographic fingerprint of SJC. 0.1 g/mL SJC extract was injected for separation on C18 reverse phase column (100×2.1mm, ACE, Scotland) on UHPLC system (Ultimate 3000, Thermo Scientific, USA). The elution condition was as follows, ACN(A)-1% Acetate acid in H2O(B): 0 min: A-B=12%-88%; 2 min: A-B=12%-88%; 11 min: A-B=28%-72%. Column temperature was 40°C, and the flow rate was 0.5 mL/min. Detection wavelength was 345 nm.

Supplementary Table 2: Genes with differential expression upon SJC treatment

Official gene symbol	Name
Down-regulated genes	
CDKN3	cyclin dependent kinase inhibitor 3(CDKN3)
BRCA2	BRCA2, DNA repair associated(BRCA2)
CTNNB1	catenin beta 1(CTNNB1)
WT1	Wilms tumor 1(WT1)
FOS	Fos proto-oncogene, AP-1 transcription factor subunit(FOS)
APC	APC, WNT signaling pathway regulator(APC)
SERPINB5	serpin family B member 5(SERPINB5)
E2F1	E2F transcription factor 1(E2F1)
RASSF1	Ras association domain family member 1(RASSF1)
Up-regulated genes	
MYCN	v-myc avian myelocytomatosis viral oncogene neuroblastoma derived homolog(MYCN)
ERBB2	erb-b2 receptor tyrosine kinase 2(ERBB2)
ETS1	ETS proto-oncogene 1, transcription factor(ETS1)
BCL2L1	BCL2 like 1(BCL2L1)
FHIT	fragile histidine triad(FHIT)

Supplementary Table 3: Detail of GO terms

GO Code	Description
GO:0045893	positive regulation of transcription, DNA-templated
GO:0008285	negative regulation of cell proliferation
GO:0007050	cell cycle arrest
GO:0043065	positive regulation of apoptotic process
GO:0001708	cell fate specification
GO:0030539	male genitalia development
GO:0045944	positive regulation of transcription from RNA polymerase II promoter
GO:1904886	beta-catenin destruction complex disassembly
GO:0001658	branching involved in ureteric bud morphogenesis
GO:0045892	negative regulation of transcription, DNA-templated
GO:0060070	canonical Wnt signaling pathway
GO:0000122	negative regulation of transcription from RNA polymerase II promoter
